# High flow nasal cannula oxygen therapy in immunocompromised patients with acute hypoxemic respiratory failure

**DOI:** 10.1186/2197-425X-3-S1-A425

**Published:** 2015-10-01

**Authors:** J-P Frat, S Ragot, C Girault, R Coudroy, R Robert, J-M Constantin, G Prat, T Boulain, A Jamet, A Mercat, L Brochard, AW Thille, for the FLORALI Study Group and REVA Network

**Affiliations:** CHU Poitiers, Service de Réanimation Médicale, Poitiers, France; Université Poitiers, INSERM CIC 1402 (Équipe 5 ALIVE), Poitiers, France; Université Poitiers, INSERM CIC 1402 Biostatistics, Poitiers, France; CHU Rouen, Service de Réanimation Médicale, Rouen, France; Institute for Biomedical Research and Innovation, Université Rouen, UPRES EA 3830-IRIB, Rouen, France; CHU Clermont Ferrand, Pôle de Médecine Périopératoire, Clermont-Ferrand, France; Université d'Auvergne, R2D2, EA-7281 Clermont-Ferrand, France; CHU de la Cavale Blanche, Service de Réanimation Médicale, Brest, France; CHR Orléans, Réanimation Médico-Chirurgicale, Orléans, France; CHU Angers, Service de Réanimation Médicale et Médecine Hyperbare, Angers, France; Keenan Research Centre and Critical Care Department, St Michael's Hospital, Toronto, Canada; Interdepartmental Division of Critical Care Medicine, University of Toronto, Toronto, Canada; Université Créteil, INSERM UMR 955 Eq13, Créteil, France

## Introduction

In the early 2000's, two randomized controlled trials have shown that non-invasive ventilation (NIV) could decrease mortality of immunocompromised patients admitted to ICU for acute respiratory failure (ARF) as compared to standard oxygen therapy (O_2_) [1, 2]. However, the benefits of NIV in immunocompetent patients with ARF failure are debated. High flow nasal cannula oxygen therapy (High-Flow Oxygen) may offer an alternative in hypoxemic patients. We recently found in a randomized controlled trial including 310 patients with ARF that High-Flow Oxygen decreased mortality as compared to NIV [3]. Immunocompromised patients could be also included in this study, except those with profound neutropenia. Therefore, we assessed the benefits of High-Flow Oxygen or NIV in this subgroup of patients.

## Objectives

To compare intubation and mortality rates in the subset of immunocompromised patients admitted to ICU for ARF.

## Methods

We performed a subgroup analysis of the FLORALI study. This study included all patients with non-hypercapnic (PaCO_2_ ≤ 45 mm Hg) ARF excluding patients with cardiogenic pulmonary edema and those with underlying chronic lung disease. Patients were assigned to three groups according to treatment: High-Flow Oxygen, O_2_ or NIV. The primary outcome was the intubation rate and secondary outcome included 90-day mortality. We focused on the subset of immunocompromised patients included in this study, knowing that patients with profound neutropenia were excluded.

## Results

Among the 310 patients with ARF, 82 (26%) were immunocompromised including 26 patients in the High-Flow Oxygen group, 30 in the O_2_ group, and 26 in the NIV group. Intubation rates were 31%, 43% and 55% in the High-Flow Oxygen, O_2_ and NIV groups, respectively (p = 0.04). The 90-day mortality rates were 15%, 27% and 46% in the High-Flow Oxygen, O_2_ and NIV groups (p = 0.046). Ventilator-free days at day 28 were 26 ± 6, 23 ± 10 and 14 ± 13 days in the High-Flow Oxygen, O_2_ and NIV groups, respectively (p < 0.0001).

## Conclusions

In immunocompromised patients admitted to ICU for acute hypoxemic respiratory failure, High-Flow Oxygen was associated with lower intubation and mortality rates, and a reduced duration of invasive mechanical ventilation as compared to O_2_ or NIV.
